# Density-dependent distributions of hosts and parasitoids resulting from density-independent dispersal rules: implications for host–parasitoid interactions and population dynamics

**DOI:** 10.1186/s40462-024-00525-2

**Published:** 2024-12-27

**Authors:** Toshinori Okuyama

**Affiliations:** Department of Entomology, National Taiwan University, Taipei, Taiwan

**Keywords:** Dispersal, Individual-based model, Aggregation, Density-dependence, Pseudointerference

## Abstract

**Background:**

The distribution of hosts and parasitoids across patches is a key factor determining the dynamics of host-parasitoid populations. To connect behavioral rules with population dynamics, it is essential to comprehend how individual-level dispersal behavior influences the distribution of individuals. Typically, a simple deterministic model has been used to describe this connection. This study explicitly derived the relationship between individual-level dispersal behavior and the distribution of individuals across patches, contrasting it with the conventional deterministic model.

**Methods:**

A stochastic individual-based model was developed from a widely used deterministic host–parasitoid population model. Individual-level dispersal rules were simulated in the stochastic model without assuming the resulting distributions. The models assume that the dispersal of hosts and parasitoids is independent of conspecific density. The deterministic model can be seen as an approximation of the stochastic model, describing the outcomes of stochastic processes as their expected patterns. In addition to describing the relationship between dispersal behavior and distribution across patches, its consequences for population dynamics were also examined.

**Results:**

The stochastic model revealed that the distribution of individuals among patches varies with the number of dispersing conspecifics, whereas the deterministic model assumes independence from conspecific density, indicating that the deterministic model fails to capture the outcomes of stochastic dispersal. The resulting density-dependent distributions of hosts and parasitoids lead to other density-dependent interactions between them, such as density-dependent parasitism risk for hosts and density-dependent searching efficiency for parasitoids, ultimately affecting population dynamics. For instance, while aggregation of parasitoids is stabilizing in the deterministic model, it can be both stabilizing and destabilizing in the stochastic model.

**Conclusions:**

The stochastic model revealed that density-dependent distributions of hosts and parasitoids emerge when individuals disperse in a density-independent manner, significantly impacting existing host-parasitoid theory, which assumes density-independent distributions. To address this, the implications of emerging density dependencies for well-known results, such as the pseudointerference of parasitoids and the CV^2^ > 1 rule, were discussed. Explicitly considering individual-level dispersal behavior is essential for understanding host–parasitoid interactions and population dynamics.

**Supplementary Information:**

The online version contains supplementary material available at 10.1186/s40462-024-00525-2.

## Background

Host-parasitoid population models typically describe individual-level behavioral and demographic processes (e.g., foraging behavior and reproduction) and scale them up to population dynamics [1, 2]. For example, many functional response models were mechanistically derived from the behavioral rules of foragers [3–5] and are a key component of population models. The dispersal of hosts and parasitoids is an individual-level behavioral rule commonly included in host-parasitoid population models [6–9]. Even when hosts and parasitoids cannot persist (i.e., hosts and/or parasitoids will eventually die out) within a single patch, they may persist through dispersal dynamics when hosts and parasitoids are patchily distributed [2].

The dispersal behavior of hosts and parasitoids influences the correlation between host and parasitoid densities across patches [10–12]. When there are $N$ patches, and $\alpha_{i}$ and $\beta_{i}$ ($i$ = 1, …, $N$) are respectively the proportions of hosts and parasitoids in patch $i$ ($\sum\nolimits_{i = 1}^{N} {\alpha_{i} = 1}$ and $\sum\nolimits_{i = 1}^{N} {\beta_{i} = 1}$), a conventional deterministic model describes the relationship between $\alpha_{i}$ and $\beta_{i}$ as

1 $$\beta_{i} = u\alpha_{i}^{\mu }$$

where $\mu$ represents host-dependent dispersal of parasitoids with $\mu = 0$ indicating “random search” [13–16], and $u$ is the normalizing constant [6, 17, 18]. The deterministic model, Eq. 1, describes the expected pattern of stochastic parasitoid dispersal. When the expected pattern is unknown, it can be revealed through stochastic simulation. For example, random search of parasitoids ($\mu$ = 0) can be simulated by randomly selecting a destination patch for each parasitoid individual, with each patch having an equal probability of being chosen. Simulating a large number of parasitoid dispersals reveals the expected distribution of parasitoids across patches; this approach is known as stochastic individual-based modeling. If the deterministic model is appropriate, the pattern predicted by Eq. 1 should match the pattern produced by the stochastic model.

One of the most influential results of host–parasitoid models is the “CV^2^ > 1 rule” where CV^2^ is the square of the coefficient of variation in parasitism risk [19, 20]. In this study, CV^2^ always refers to this quantity. The rule states that when the inequality condition is satisfied, a large class of host–parasitoid systems is stable. The parameter $\mu$, which describes host-dependent parasitoid dispersal, increases CV^2^ by introducing greater variability in parasitoid densities across patches [21]. In the CV^2^ > 1 rule, CV^2^ is assumed to be a constant property of host–parasitoid systems, derived from the assumption that the expected proportions of hosts and parasitoids, $\alpha_{i}$ and $\beta_{i}$, are constant parameters (with $\beta_{i}$ constant because $\mu$ is a constant parameter) in the deterministic model. Visser et al. [22], in a laboratory experiment, provided patches that contained the same number of hosts to varying number of parasitoids and found that the distribution of parasitoid effort among patches became more uniform as the number of parasitoids increased, violating the assumption of conspecific-density-independent distribution of parasitoids. The authors discussed that this density-dependence may be due to conspecific-density-dependent behavioral rules (e.g., $\mu$ in Eq. 1 changes with parasitoid density) [22]. While such conspecific-density-dependent behavioral rules may be important, we must first understand the expected distribution of parasitoids in the absence of conspecific-density-dependent behavioral rules in order to evaluate the role of conspecific-density-dependent behavioral rules.

Despite the importance of the relationship between dispersal behavior and resulting distribution of individuals among patches, the relationship has not been carefully evaluated, while a simple model such as Eq. 1 has been routinely used. Understanding the result of conspecific-density-independent dispersal of hosts and parasitoids is particularly important because it becomes the reference of comparison to evaluate more complex density-dependent dispersal rules as discussed above. This study used an individual-based modeling approach, as discussed above, and shows that the deterministic model fails to capture patterns produced by stochastic dispersal of hosts and parasitoids. The importance of this issue in interpreting existing results, such as the CV^2^ > 1 rule and the observed conspecific-density-dependent distribution of parasitoids, is discussed. Hereafter, unless otherwise specified, density dependence and independence always refer to conspecific density, and the word 'conspecific' will not be explicitly stated.

## Methods

### Deterministic model

The model describes a situation in which hosts and parasitoids (one species each) are distributed in $N$ distinct patches. $H_{t}$ and $P_{t}$, respectively, are the total number of hosts and parasitoids at beginning of *t*th generation. The population dynamics are described by

2 $$H_{t + 1} = \lambda H_{t} \mathop \sum \limits_{i = 1}^{N} \alpha_{i} {\text{exp}}\left( { - a\beta_{i} P_{t} } \right)$$

3 $$P_{t + 1} = cH_{t} \left( {1 - \mathop \sum \limits_{i = 1}^{N} \alpha_{i} {\text{exp}}\left( { - a\beta_{i} P_{t} } \right)} \right)$$

where $\lambda$ is the growth rate of the host, $a$ is the searching efficiency of the parasitoid, and $c$ is the number of parasitoids emerging from a parasitized host. The interpretations of $\alpha_{i}$ and $\beta_{i}$ as well as their relationship $\beta_{i} = u\alpha_{i}^{\mu }$ (Eq. 1) follow those described above. When $\mu = 0$ the distribution of parasitoids is independent of host densities, $\mu = 1$ indicates the distributions of hosts and parasitoids are the same, $\alpha_{i} = \beta_{i}$, and as $\mu$ increases further, parasitoids increasingly aggregate in patches of relatively high density. This deterministic model has been analyzed in details [6, 13, 14]. In relation to the CV^2^ > 1 rule, $\mu$ and CV^2^ are positively related, and an unstable system can be stabilized by increasing $\mu$ [6].

### Stochastic model

The deterministic model was translated into a stochastic individual-based model, referred to as the stochastic model in this study. As discussed in the introduction, the parameter $\mu$ (Eq. 1) is commonly defined in terms of host-dependent parasitoid dispersal such that $\mu = 0$ indicates “random search” [13–15]. By following the behavioral interpretation, ${\varvec{\alpha}} = (\alpha_{1} ,\alpha_{2} , \cdots ,\alpha_{N} )$ and ${\varvec{\beta}} = (\beta_{1} ,\beta_{2} , \cdots ,\beta_{N} )$ are interpreted as the probabilities that a host and parasitoid disperses into patch $i$. The model is described as a pseudocode for clarity [23]. The code outlines the processes occurring in a generation, where the total numbers of hosts and parasitoids in the generation are denoted by $H$ and $P$.

#### Pseudocode

1. [Host dispersal] Generate a random vector from a multinomial distribution with size $H$ and probability vector ${\varvec{\alpha}}$. Let the $i$ th element of the random vector be $h_{i}$ representing the number of hosts dispersed to patch $i$.
2. [Parasitoid dispersal] Generate a random vector from a multinomial distribution with size $P$ and probability vector ${\varvec{\beta}}$ computed by the actual distribution of hosts: $\beta_{i} = uh_{i}^{\mu }$. Let the $i$ th element of the random vector be $p_{i}$ representing the number of parasitoids dispersed to patch $i$.
3. [Parasitism] In patch $i$ (for all patches), generate a random number from a binomial distribution with size parameter $h_{i}$ and probability parameter ${\text{exp}}\left( { - ap_{i} } \right)$. This random number represents the number of hosts escaped parasitism in patch $i$, $h_{i}^{{{\text{esc}}}}$. Consequently, the number of hosts that are parasitized is $h_{{\text{i}}} - h_{{\text{i}}}^{{{\text{esc}}}}$.
4. [Hosts and parasitoids for the next generation] The total number of parasitoids contributed from this generation to the next generation is the total number of parasitized hosts, $\sum\nolimits_{i = 1}^{N} {\left( {h_{{\text{i}}} - h_{{\text{i}}}^{{{\text{esc}}}} } \right)}$ for solitary parasitoids. The total number of hosts contributed from this generation to the next generation is simulated as a random number from a Poisson distribution with mean $\lambda \sum\nolimits_{i = 1}^{N} {h_{i}^{{{\text{esc}}}} }$. To study population dynamics, repeat from step 1 with the updated counts of hosts and parasitoids.

### Analysis

#### Stochastic dispersal

The effect of stochastic dispersal on the proportions of individuals among patches is described. Because dispersal is simulated by multinomial distributions, this investigation simply describes patterns of multinomial outcomes. Although this is an individual-based model, a multinomial distribution is used to simulate the dispersal of all individuals at once. This approach yields the same result as simulating dispersal individually. The analysis focuses on random dispersal of hosts ($\alpha_{i} = 1/N$ for all patches) and parasitoids. Parasitoids disperse randomly when they exhibit host-independent dispersal ($\mu$ = 0) or when the number of hosts is the same for all patches.

#### Per-capita parasitism risk

The relationship between per-capita parasitism risk and the total number of hosts in the stochastic model was investigated. The per-capita parasitism risk was defined as the average parasitism probability calculated across all hosts in $N$ patches. Specifically, it is given by $\sum\nolimits_{i = 1}^{N} {(h_{{\text{i}}} - h_{{\text{i}}}^{{{\text{esc}}}} )/H}$ where $H$ is the total number of hosts. This analysis describes how parasitism efficiency (in terms of per-capita parasitism risk) changes with the number of hosts while fixing the number of parasitoids.

#### Overall search rate

The overall search rate $s$ is the per-capita searching efficiency of parasitoids, computed for the whole population, and is calculated as,

4 $$s = \frac{1}{P}{\text{log}}\left( {\frac{H}{{H - H_{a} }}} \right)$$

where *H* and *P* are the number of hosts and parasitoids, respectively, and $H_{a}$ is the number of hosts parasitized in the generation [13, 14, 24]. All variables are total numbers summed across all patches; for example, $H_{a} = \sum\nolimits_{i = 1}^{N} {\left( {h_{i} - h_{i}^{{{\text{esc}}}} } \right)}$. In the deterministic model, this quantity decreases as the parasitoid density increases when $\alpha_{i}$ is not the same for all patches and host-dependent dispersal of parasitoid $\mu$ is not zero. This decrease in $s$ with an increase in parasitoid density is referred to as pseudointerference [14]. It is termed pseudointerference because, despite the absence of explicit parasitoid-parasitoid interference in the model, parasitoid performance decreases with increasing parasitoid density, mimicking the effects of interference.

#### Population dynamics

The effect of host-dependent dispersal of parasitoids $\mu$ on the stability of the stochastic model was examined. In the stochastic model, equilibrium stability is not attained due to stochastic fluctuation. Therefore, ‘persistence’ was used as a measure of stability. If both the host and parasitoid populations persist (i.e., the population sizes do not become zero) for 1000 generations, it was considered a successful persistence. The initial densities of the stochastic model were derived from the equilibrium densities of the deterministic model. The equilibrium densities of parasitoids and hosts in one patch model ($N$ = 1) are $p^{*} = {\text{ln}}\left( \lambda \right)/a$ and $h^{*} = \left( {\lambda /\left( {\lambda - 1} \right)} \right)\left( {p^{*} /c} \right)$. The initial densities of the stochastic models were $P_{0} = \mu^{1.2} Np^{*}$ and $H_{0} = \left( {\lambda /\left( {\lambda - 1} \right)} \right)(P_{0} /c)$ (both values were rounded up). In the stochastic model, population densities can widely fluctuate, and the average densities increase with host-dependent dispersal of parasitoids $\mu$. An important consideration is that a persisting system (i.e., a system that persists with the probability of one) may never persist for some initial densities. In an extreme example, all hosts will be parasitized in the initial generation if the number of parasitoids in the generation is indefinitely increased in any persisting system. Initial densities should be close to a realization of potentially persisting dynamics, and the initial densities described above are sufficient for the ranges of parameters considered. The dynamics of CV^2^ in relation to population dynamics were also described.

#### Parameters

The parameter definitions of the models and their corresponding values used in the analysis are shown in Table 1. For all analyses, a solitary parasitoid ($c$ = 1) and random dispersal of hosts ($\alpha_{i} = 1/N$ for all patches) were assumed. Random dispersal of hosts represents situations where the quality of all patches is equivalent for the hosts. The number of patches ($N$), host growth rate ($\lambda$), and host-density-dependent dispersal of parasitoids ($\mu$) were varied widely to encompass a broad range of host–parasitoid systems. For simplicity, the parasitoid searching efficiency ($a$ = 1) was used. However, this parameter is confounded with the number of parasitoids ($P$) in the model, such that $a$ = 1 and $P$ = 10, and $a$ = 0.1 and $P$ = 100 yield the same expectation as $aP$ describes the parasitism risk (Eqs. 2–3). The number of parasitoids was varied in the analysis examining the parasitism pattern occurring within a single generation. The base code for the stochastic model in R [25] is provided in Supplementary Information.

**Table 1:** Parameters and their values used in the analysis are listed. Not all parameters are relevant to all analyses. The analysis of stochastic dispersal examines only random dispersal

Symbol	Definition	Value
$a$	Parasitoid searching efficiency	1
$\lambda$	Host growth rate	2 to 12
$c$	Number of emerging parasitoids per parasitized host	1
$N$	Number of patches	10, 100, 1000
$\alpha_{i}$	Host dispersal probability to patch $i$	$1/N$
$\mu$	Host-dependent parasitoid dispersal	0 to 40

## Results

### Stochastic dispersal

**Fig. 1:**
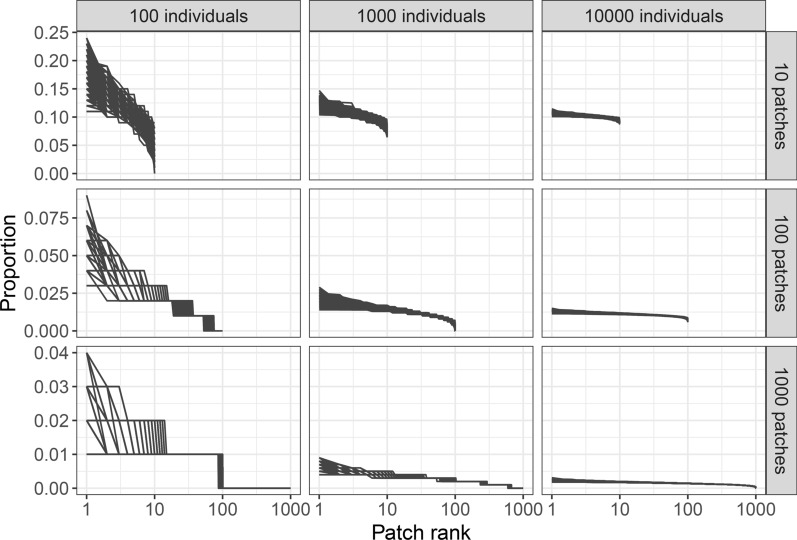
Proportion of individuals in patches as a result of random dispersal. If dispersing individuals are hosts, $\alpha_{i} = 1/N$; if parasitoids, $\mu$ = 0, or the number of hosts is the same for all patches. The expectation from the deterministic model is 1/$N$ for all $N$ patches. The results of the stochastic dispersal become closer to the deterministic expectation as the number of dispersing individuals increases for a given number of patches. Each line shows the outcome of a simulation, with results from 10,000 simulations displayed together for each combination of the number of patches $N$ and the number of dispersing individuals. Patches are ranked according to the relative proportion (i.e., rank 1 is the patch with the largest number of individuals)

When individuals randomly disperse into $N$ patches, the resulting proportions of individuals among patches are skewed by expectation (Fig. 1). The result is applicable to any randomly dispersing individuals, whether they are hosts ($\alpha_{i} = 1/N$ for all patches) or parasitoids (e.g., $\mu = 0$). The expected distribution of the stochastic model becomes closer to the expectation of the deterministic model (i.e., uniform distribution) as the number of dispersing individuals relative to the number of patches increases (Fig. 1). Although only random dispersal is shown here, expected rank proportions become closer to the underlying parameters (i.e., ranked ${\varvec{\alpha}}$ and ${\varvec{\beta}}$) as the number of dispersing individuals increase even when dispersal is non-random. The population model (Eqs. 2–3) considers situations where dispersal occurs only once at the beginning of each generation. If individuals disperse multiple times within a generation according to the same behavioral rule, it has the similar effect as increasing the number of individuals.

### Per-capita parasitism risk

**Fig. 2:**
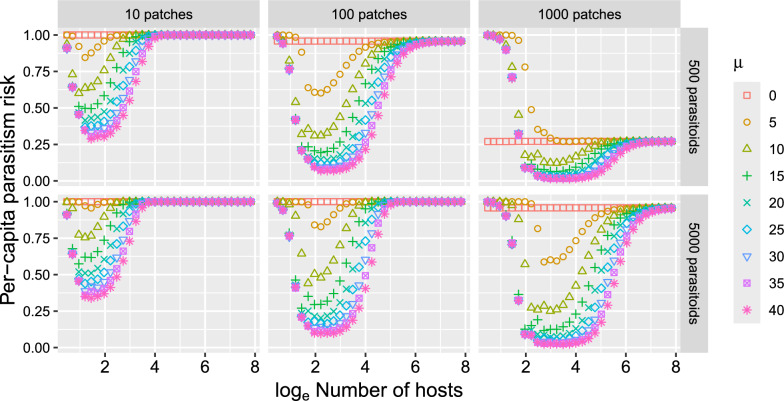
Relationship between per-capita parasitism risk and the number of hosts. The per-capita parasitism risk varies with the number of hosts when parasitoids exhibit host-dependent dispersal, whereas the corresponding deterministic model predicts that it is independent of the number of hosts. The number of patches $N$, number of parasitoids $P$, and host-dependent parasitoid dispersal $\mu$ as were varied as shown in the figure. $\alpha_{i} = 1/N$ for all *i*, and, *a* = 1. Each value presented in the figure is the average of 10,000 simulations

In the stochastic model, the per-capita parasitism risk of hosts depends on the number of hosts when parasitoid dispersal is not random (Fig. 2). In the corresponding deterministic model, where $\alpha_{i} = 1/N$, $\mu$ has no effect, and per-capita parasitism risk is independent of the number of hosts. When parasitoids disperse in a host-dependent manner, the per-capita parasitism risk is generally lowest at intermediate host levels. From this lowest point, per-capita parasitism risk increases as the number of hosts either increases or decreases.

### Overall search rate

**Fig. 3:**
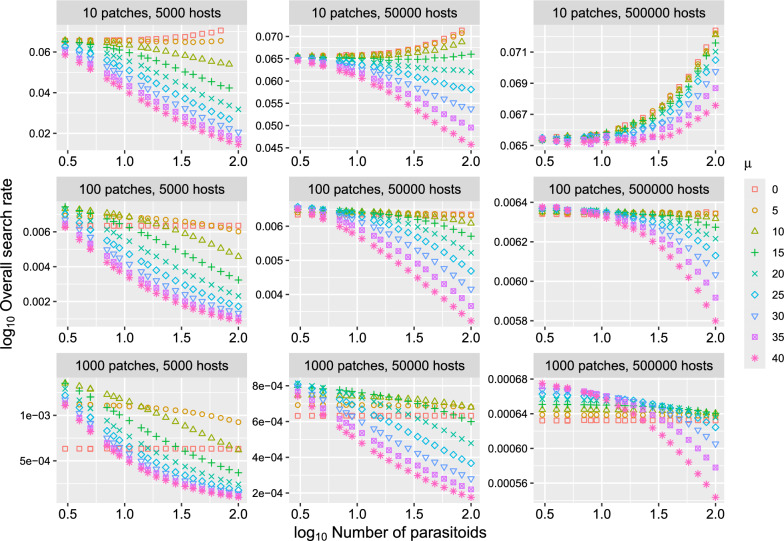
Relationship between overall search rate $s$ and the number of parasitoids $P$ for various combinations of the number of patches ($N$), number of hosts ($H$) and host-dependent parasitoid dispersal ($\mu$). The overall search rate varies with the number of parasitoids in the stochastic model, while the corresponding deterministic model predicts that it is constant. $\alpha_{i} = 1/N$ for all *i* and *a* = 1. Values shown in the plots are averages from 10,000 simulations. Some combinations show no values because all hosts were parasitized at least in one simulation

The overall search rate of parasitoids may be positively or negatively influenced by the number of parasitoids in the stochastic model (Fig. 3). The corresponding deterministic model predicts that the overall search rate is independent of the number of parasitoids and other parameters because host dispersal is random ($\alpha_{i} = 1/N$). In addition to pseudointerference (i.e., the negative effect of parasitoid density on overall search rate), positive effects of parasitoid density on search rate, which may be termed pseudofacilitation, is also observed when the number of hosts is much higher than the number of patches and host-dependent dispersal of parasitoid is weak ($N$ = 10 in Fig. 3).

#### Population dynamics

**Fig. 4:**
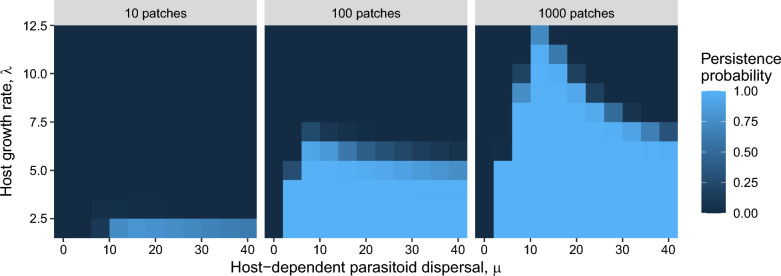
Probability of persistence associated with different combinations of the number of patches ($N$), host growth rate ($\lambda$), and host-dependent parasitoid dispersal ($\mu$). The deterministic model predicts that persistence is never possible when host dispersal is random, but it is possible in the stochastic model as shown. The parameter combinations resulting in persistence increase as the number of patches increases. $\alpha_{i} = 1/N$ for all *i*, and $a$ = 1. The persistence probability in each parameter combination was estimated based on 10,000 simulations

Persistence is possible for some parameter combinations in the stochastic model (Fig. 4), whereas persistence is impossible for any parameter combinations in the corresponding deterministic model with $\alpha_{i}$ = 1/$N$. Generally, $\mu$ stabilizes population dynamics as very low values ($\mu$ $\approx$ 0) never result in persistence, and an increase in $\mu$ makes persistence possible as long as the host growth rate is not too high. However, $\mu$ can also be destabilizing as the persistence probability can decrease by further increasing $\mu$. Although this destabilizing effect of $\mu$ is observed in all patch numbers examined, it is most pronounced when the number of patches ($N$) and host growth rate ($\lambda$) are high. This destabilizing effect of $\mu$ is not observed in the deterministic model even when the assumption of $\alpha_{i}$ = 1/$N$ is relaxed.

**Fig. 5:**
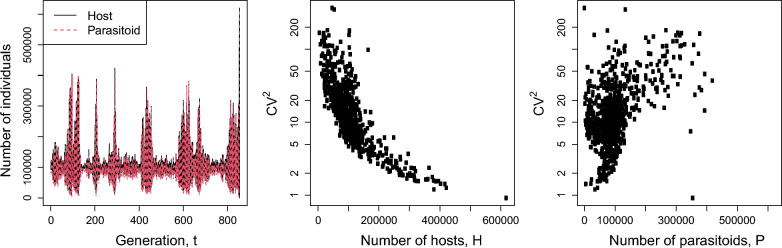
Realized dynamics of populations and CV^2^. $\alpha_{i} = 1/N$ for all *i*, *a* = 1, $N$ = 1000, $\mu$ = 20, and $\lambda$ = 10. Extinction of hosts occurred at 855th generation

A realization of non-persistent population dynamics resulting from an increase in $\mu$ is shown in Fig. 5. There is negative correlation between CV^2^ and host density. The relationship between CV^2^ and parasitoid density is weak. Non-persistent dynamics experience outbreaks of populations that can lead to extinction. Such outbreaks are partially triggered by host density-dependent CV^2^ as high values of CV^2^ indicates opportunities for a rapid population growth when $\lambda$ is high.

## Discussion

Stochastic dispersal induces density-dependent skewness in the proportions of individuals among patches in the absence of any density-dependent behavioral rules (Fig. 1). This density-dependent skewness leads to other density dependencies such as density-dependent per-capita parasitism risk (Fig. 2), overall search rate (Fig. 3), and CV^2^ (Fig. 5). Since the density-dependent distribution of hosts and parasitoids emerges through fundamental statistical processes, the results described in this study are relevant to any host–parasitoid system.

Regardless of whether hosts and parasitoids disperse randomly or non-randomly, the resulting expected rank-abundance is density-dependent and does not follow ${\varvec{\alpha}} = (\alpha_{1} ,\alpha_{2} , \cdots ,\alpha_{N} )$ or ${\varvec{\beta}} = (\beta_{1} ,\beta_{2} , \cdots ,\beta_{N} )$. The expected proportion of hosts in patch $i$ is $\alpha_{i}$ when $\alpha_{i}$ is the probability of dispersing to patch $i$. However, this expectation is not relevant to host–parasitoid interactions; the important detail is the relative abundance of hosts in patches. Realizations of multinomial elements are not independent, and thus we cannot predict relative abundance by focusing on each patch independently. For example, when 10 hosts disperse randomly across two patches and 7 hosts end up in the first patch by chance, it automatically means that 3 hosts end up in the second patch. To understand the relative proportions of individuals in the patches, all patches must be considered simultaneously.

Density-dependent per-capita parasitism risk in hosts (Fig. 2) and density-dependent CV^2^ (Fig. 5) result from density-dependent distribution in hosts (Fig. 1). As the host density increases, the distribution of hosts become closer to uniform, which reduces the aggregation of parasitoids and enhances the parasitism efficiency of the parasitoid population, producing negative density-dependence in CV^2^ (Fig. 5) and positive density-dependence in per-capita parasitism risk (Fig. 2). When host density is sufficiently low relative to the number of patches, negative density-dependence in per-capita parasitism risk was also observed (Fig. 2). As the number of hosts becomes fewer, the number of empty patches increases (Fig. 1). Because the model assumes that parasitoids do not disperse to empty patches when $\mu$ > 0 (Eq. 1), this reduction in number of patches enhances the parasitism efficiency of the parasitoid population. This detail is unique in the individual-based model, as in the deterministic model (Eqs. 2–3), the density 1/$H$ of hosts disperses to all $N$ patches regardless of the values of $H$ and $N$.

Density-dependent overall search rate (Fig. 3) can be explained by density-dependent distribution of parasitoids (Fig. 1). While the negative effect of parasitoid density on overall search rate is a well-recognized effect (i.e., pseudointerference), positive effects of parasitoid density on overall search rate were also observed when the number of hosts are significantly larger than the number of patches. For example, when $N$ = 10 and $H$ = 500,000, the relationships were entirely positive (Fig. 3). This is because the distribution of hosts is close to a uniform distribution such that the effect of $\mu$ becomes weak, resulting in the random dispersal of parasitoids. In other words, as parasitoid density-increases, the distribution of parasitoids also approaches to a uniform distribution, which increases the overall search rate of parasitoids.

Although the negative relationship between parasitoid density and the overall search rate of parasitoids is well known as pseudointerference, another mechanism called indirect mutual interference can also produce a similar relationship. Indirect mutual interference is a decrease in searching efficiency, detectable at the population level, mediated by conspecific-dependent behavior that is not related to direct interference [26]. The model examined in this study contains no component of indirect mutual interference because the dispersal behavior of parasitoids does not change with parasitoid density. However, when dispersal behavior changes with parasitoid density (e.g., $\mu$ in Eq. 1 changes with parasitoid density), it can induce indirect mutual interference. To differentiate pseudointerference from indirect mutual interference, we must understand the distribution of parasitoids in the absence of density-dependent behavior. It is important to remember that a density-dependent distribution can result from density-independent behavioral rules (Fig. 1). If we mistakenly interpret an observed density-dependent distribution of parasitoids as a result of density-dependent behavioral rules, we may falsely detect indirect mutual interference, even when it does not exist.

The parameters $\alpha_{i}$ and $\beta_{i}$ represent different concepts in the deterministic and stochastic models. In the stochastic model, they correspond to individual behavioral rules, whereas in the deterministic model, $\alpha_{i}$ and $\beta_{i}$ represent the population-level patterns (i.e., the outcomes of behavioral processes). However, the interpretations in the deterministic models commonly mix both behavioral and population level interpretations such as $\mu$ = 0 indicating the random search of parasitoids as discussed above. If random dispersal were to be described within the framework of the deterministic model, $\alpha_{i}$ and $\beta_{i}$ should represent the expected rank abundance that is density-dependent (Fig. 1). However, such expected rank abundance from a multinomial distribution is unknown. If we interpret $\alpha_{i}$ and $\beta_{i}$ strictly as the distributions within the deterministic model, there may be no interpretation issue. Still, a mechanism is needed to explain why $\alpha_{i}$ and $\beta_{i}$​ are constants, as density-independent distributions are unlikely if individual dispersal rules are density-independent. One might argue for optimal foraging and its expectation, but the results of this study show that how closely an expectation is realized depends on the density of host and parasitoid.

This study highlights the usefulness of a stochastic individual-based approach. The deterministic model assumes the distributions of host and parasitoids as constant parameters (Eqs. 2–3), which also assumes CV^2^ is constant. In modeling studies, CV^2^ is introduced as a model parameter to examine the effect of CV^2^ on population dynamics [27, 28]. However, the stochastic model shows all these quantities, commonly assumed as constant parameters, are actually density-dependent variables (Figs. 1 and 5). Consequently, the statement that ‘CV^2^ is a stabilizing factor’ loses a clear interpretation when it is not a constant parameter of a system but rather a density-dependent variable. Given that CV^2^ is density-dependent, its effect on population dynamics likely depends on the nature of this density-dependence, influenced by the individual behavior of hosts and parasitoids, which may be explored in future studies. Density-dependence is a key element of population dynamics, and much research effort has been devoted to identifying and describing various types of density dependences [29–32]. The assumption of the absence of density dependences in the deterministic models must be carefully interpreted.

## Conclusions

The distributions of hosts and parasitoids across patches are conventionally assumed to be fixed constants, even though fundamental statistical processes make them density-dependent. Properly understanding density dependence is crucial for interpreting observed density-dependent patterns. For example, a system without indirect mutual interference might be misidentified as having indirect mutual interference if density-dependent distribution changes are assumed to result only from density-dependent behavioral rules. Similarly, an increase in per-capita parasitism risk with host density (Fig. 2) might be misinterpreted as a type III functional response of the parasitoid (e.g., parasitoids become acceleratingly more efficient at finding hosts as host density rises). Qualitatively similar density-dependent patterns can arise from multiple mechanisms. Careful and explicit consideration of stochastic dispersal can prevent the misinterpretation of observed patterns and help identify previously unrecognized factors contributing to the stability of host–parasitoid population dynamics.

## Supplementary Information

Additional file 1.

## Data Availability

The core code used in the study is available in Supplementary Information.
